# Eye tracking in human interaction: Possibilities and limitations

**DOI:** 10.3758/s13428-020-01517-x

**Published:** 2021-01-06

**Authors:** Niilo V. Valtakari, Ignace T. C. Hooge, Charlotte Viktorsson, Pär Nyström, Terje Falck-Ytter, Roy S. Hessels

**Affiliations:** 1 Experimental Psychology, Helmholtz Institute, Utrecht University, Heidelberglaan 1, 3584 CS Utrecht, the Netherlands; 2 Department of Psychology, Uppsala University, Uppsala, Sweden; 3 Karolinska Institutet Center of Neurodevelopmental Disorders (KIND), Department of Women’s and Children’s Health, Karolinska Institutet, Stockholm, Sweden; 4 Swedish Collegium for Advanced Study (SCAS), Uppsala, Sweden

**Keywords:** Eye tracking, Human interaction, Data analysis, Data quality, Wearable

## Abstract

There is a long history of interest in looking behavior during human interaction. With the advance of (wearable) video-based eye trackers, it has become possible to measure gaze during many different interactions. We outline the different types of eye-tracking setups that currently exist to investigate gaze during interaction. The setups differ mainly with regard to the nature of the eye-tracking signal (head- or world-centered) and the freedom of movement allowed for the participants. These features place constraints on the research questions that can be answered about human interaction. We end with a decision tree to help researchers judge the appropriateness of specific setups.

## Introduction

Human interaction, which we here define as action occurring between (at least) two individuals, has intrigued researchers and philosophers across many different disciplines for a long time (e.g., Argyle & Dean, 1965; Duncan & Fiske, 1977; Goffman, 1955; Kendon, 1967). Human interaction is multimodal, and may include vision, audition, and haptics. In this article, our main focus will be on techniques to measure gaze, i.e., where one looks and when, as it often has an important role in face-to-face interaction (see Brône & Oben, 2018; Hessels, 2020; Land & Tatler, 2009 for extensive overviews of looking behavior in human interaction). Humans are foveated animals, which means that visual acuity is greatest at the center of the retina, the fovea; objects close to the gaze location are represented in higher resolution than those in the periphery (see e.g., Yarbus, 1967 for an overview of the structure of the human eye). As such, looking at objects in the visual world can facilitate perception. In interaction, the ability to gaze around with the eyes is not only useful when attending to someone or something and scanning the environment, but it may also signal information (e.g., Gobel, Kim, & Richardson, 2015; Wu, Bischof, & Kingstone, 2014). This is evident when one considers that the body, head, and eyes of a person are commonly visible to other people in interactive situations. The orientation of the body and head and the position of the pupil and iris within the white sclera of the eye allow observers to easily estimate the gaze direction of others (e.g., Gibson & Pick, 1963; Langton, Watt, & Bruce, 2000). Take an interactive situation between a parent and an infant for example: if the infant looks intensely at a toy out of reach, the parent can see the gaze of the infant and hand the toy to the infant to play with. The gaze of the infant, as an integral part of the behavior of the infant, then apparently influenced the action of the parent.

Interest in gaze behavior during interaction has a long history. Empirical work on the posited dual role (i.e., both information acquisition and signaling) of gaze in interaction has been conducted at least since the 1960s. One exemplary study of human interaction is that by Kendon (1967). He designed an experiment to investigate the role of gaze during conversation. He instructed participant dyads to “get to know one another” (p. 24) in 30 min and videotaped the conversations and subsequently estimated the direction of their gaze from the video recordings. He found that participant dyads showed highly correlated gaze patterns (i.e., the duration of gazing at each other and rate of change of gaze direction), and that participants had a tendency to look away from their partner when beginning an utterance and back toward them when the utterance was close to its end. This type of early research on looking behavior in interaction mostly relied on using an external observer to provide an estimate of where someone is looking (see Argyle & Cook, 1976; Goodwin, 1981 for other examples of early interaction studies). With advances in technology, there has been an emergence of new methods to measure gaze more accurately and objectively, using devices commonly known as eye trackers (see Jongerius, Hessels, Romijn, Smets, & Hillen, 2020 for a review of the methods used to study eye contact).

Interest in using eye tracking to measure gaze has increased over the past years. Reviews have been written on the breadth of eye-tracking research (Duchowski, 2002), on differences between eye-tracking “in the wild” (i.e., with unrestricted movement of the body and head) and in the lab (i.e., with restricted movement of the body and head) (Lappi, 2015), and on eye movements in various situations (e.g., Hayhoe & Ballard, 2005, 2014). However, to the best of our knowledge, the possibilities and limitations of eye tracking in relation to the study of face-to-face human interaction have not yet been summarized in a structured way.^1^ The main goals of this review are (1) to describe the different types of eye-tracking setups that have been used to investigate human interaction so far, (2) to provide a scoping overview of the eye-tracking research that has been conducted on human interaction, and (3) to guide researchers into choosing the optimal setup by identifying and discussing the constraints that eye-tracking setups place on the research questions that can be asked about human interaction. With these three aims put together, our objective is to provide a structured overview of the study of looking behavior in human interaction, aimed particularly at researchers starting out in the field.

## Eye tracking

Before we discuss eye tracking in the context of human interaction, we first introduce the principles behind eye tracking. Eye trackers are devices that can be used to estimate the gaze direction of a person. Most modern video-based eye trackers are equipped with one or two cameras and one or more infrared light sources. The infrared light illuminates the eyes and face creating a corneal reflection (CR), while the camera films the eyes and the face. Eye trackers compute the point of regard (i.e., the point where gaze is directed at, such as a location on a computer screen) by comparing the location of the pupil in the camera image to the location of the CR in the camera image (Holmqvist et al., 2011). Generally speaking, an eye-tracking experiment minimally involves an eye tracker and a participant. The type, model, and number of eye trackers used varies between experiments, as does the placement of the eye tracker in relation to the participant. The overall combination of eye tracker and participant placement is what we hereafter refer to as an *eye-tracking setup*. Next, we will discuss the different types of eye-tracking setups that have been used to investigate looking behavior in face-to-face human interaction. As this is a review of eye tracking in human interaction, we do not provide a detailed discussion of the general problems faced in eye-tracking research. Readers interested in the more general aspects of eye-tracking research are referred to Holmqvist et al. (2011) or Holmqvist and Andersson (2017) for good starting points.

**Fig. 1:**
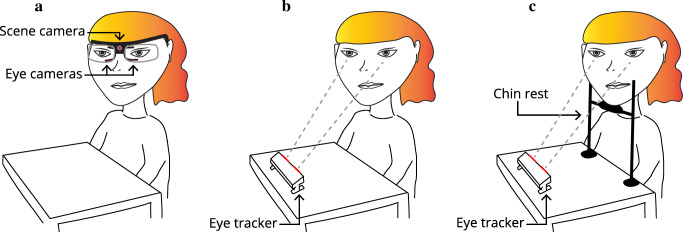
An illustration of the different types of eye-tracking setups: **a** An example of a head-free setup containing a wearable eye tracker. **b** An example of a head-boxed setup containing a remote eye tracker. **c** An example of a head-restricted setup containing a remote eye tracker and a chin rest

The types of eye-tracking setups used in interaction research can be divided into three categories based on the freedom for the participant and nature of the eye-tracking signal (see Fig. 1 for an illustration of these categories): (1) Setups with unrestrained head and an eye-tracking signal with a reference frame attached to the head (Fig. 1a). These setups will be referred to as *head-free* setups. An example of a head-free setup may contain one or more wearable eye trackers. Wearable eye trackers are mounted on the head of the participant, and usually mimic the appearance of glasses. They are typically equipped with a scene camera to provide a view of what the wearer is looking at. Early examples of wearable eye-tracking studies are given by Land, Mennie, and Rusted (1999), who had participants make a cup of tea while wearing an eye tracker, and Pelz and Canosa (2001), who had participants perform tasks that required unrestricted movement, such as walking to a washroom to wash and dry their hands. (2) Setups with an unrestrained head and an eye tracker signal that has a reference frame that is attached to the world (Fig. 1b). We will refer to these setups as *head-boxed setup*s. An example of a head-boxed setup may contain one or more remote eye trackers. Remote eye trackers are typically placed at a fixed location in front of the participant (see Merchant, Morrissette, & Porterfield, 1974 for an example of the first remote eye tracker that allowed for movement of the head). (3) Setups with a restrained head and an eye-tracking signal that has a reference frame attached to the world (Fig. 1c). These we will refer to as *head-restricted setup*s. A typical example of a head-restricted setup contains one or more tower-mounted eye trackers or remote eye trackers with a chin rest. Tower-mounted eye trackers restrict both the chin and the head and film the eyes from above (see Fig. 1c in Holleman, Hooge, Kemner, & Hessels, 2020 for a picture of a typical tower-mounted eye tracker).

In addition to the distinction between head-free, head-boxed, and head-restricted, eye-tracking setups found in the interaction literature can further be divided into two additional categories: single and dual eye-tracking setups. With single eye-tracking setups we refer to setups where the gaze of only one of the participants in an interactive situation is recorded using an eye tracker. Dual eye-tracking setups, on the other hand, allow for the simultaneous recording of the gaze of two participants using eye trackers. Table 1 contains and overview of the face-to-face interaction studies we have reviewed categorized by the type of eye-tracking setup and whether it was single or dual. The table contains as many relevant studies for each category as we are aware of. Some categories contain fewer examples as they have been less popular (although not necessarily less effective or efficient) choices for eye-tracking setups in interaction research. We make a further division between head-boxed setups with and without screens, as this choice is likely to have implications on e.g., the questions that can be asked with the setup and the ease of data analysis. We will return to this point in the section dedicated to head-boxed setups. The division of setups with and without a screen has not been made for head-restricted setups as we are only aware of studies where screens have been used.

**Table 1:** Examples of interaction studies categorized by the type of eye-tracking setup and whether it was single or dual. Studies are ordered chronologically. Note that we have separated studies using head-boxed setups based on whether they use screens for reasons explained in the main text

	Head-free	Head-boxed with screen	Head-boxed without screen	Head-restricted
Single	Gullberg and Holmqvist (1999) Gullberg and Holmqvist (2006) Hanna and Brennan (2007) Nadig et al. (2010) Franchak et al. (2011) Damm et al. (2013) Freeth et al. (2013) Macdonald and Tatler (2013) Cañigueral et al. (2018) Freeth and Bugembe (2019) Fu et al. (2019) Yamamoto et al. (2019) Haensel et al. (2020)	Merin et al. (2007) von dem Hagen and Bright (2017)	Haith et al. (1977) Gredebäck et al. (2010) Falck-Ytter (2015) Thorup et al. (2016) Nyström et al. (2017) Thorup et al. (2018) Nyström et al. (2019)	Holleman, Hessels, et al. (2020)
Dual	Broz et al. (2012) Yu and Smith (2013) Ho et al. (2015) Yu and Smith (2016) Macdonald and Tatler (2018) Franchak et al. (2018) Rogers et al. (2018) Yu et al. (2019) Cañigueral et al. (2020)	Hessels et al. (2017) Hessels, Holleman, et al. (2018) Hessels et al. (2019)		Nakamura et al. (2017)

The concept of single and dual eye-tracking setups can be expanded to setups containing even more eye trackers, which could then be referred to as triple setups, quadruple setups, and so on. Adding a new eye tracker to a setup will typically bring increased complexity to how the setup operates (e.g., in terms of required connections, computing power, and synchronization between components) and may affect how the eye-tracking data it provides is analyzed. It is, however, important to note that this might not always be the case. For example, in studies where the synchronicity of measures between participants is not of interest or in studies where the same global eye-tracking measures are used for every participant, adding another eye tracker is not likely to complicate data analysis. An example of global eye-tracking measures can be found in a study by Dowiasch, Marx, Einhäuser, and Bremmer (2015), where the saccade and eye blink frequencies made by the participants were calculated and compared across two different conditions. These types of frequency measures are global in the sense that they can be computed independent from the signals of other eye trackers or the visual stimulus. In cases where the synchronicity between the gaze signals of two or more participants is of concern, the increase in complexity is already evident when moving from a single eye-tracking setup to a dual eye-tracking setup. This might explain why, to date, the majority of eye-tracking research in human interaction has been done using setups where two people interact and with one or two eye trackers at maximum. For this reason, we will focus mainly on single and dual setups. However, the same principles apply to most multi eye-tracking setups used for studying human interaction.

## Single and dual eye-tracking setups for the study of human interaction

The choice of eye-tracking setup is closely tied to the research question. It would not make sense to investigate how people look at others during locomotion using head-boxed or head-restricted setups as such movement would require them to move out of the recording space (see e.g., Franchak, Kretch, & Adolph, 2018; Franchak, Kretch, Soska, & Adolph, 2011; Hessels, van Doorn, Benjamins, Holleman, & Hooge, 2020 who investigated looking behavior to others while locomotion could or would occur). The studies we reviewed were mostly concerned with whether gaze was directed at specific areas of interest (AOIs) and for how long, e.g., whether participants looked at the face of the other or whether some facial features were gazed at more than others. We will therefore base our discussion on the assumption that AOI-based measures are used. 

Single eye-tracking setups are generally used for the analysis of a single person's gaze data (note that there are exceptions to this, as we will point out in the following section). Gaze-based measures relevant to human interaction that can be obtained with single eye-tracking setups might include the number and duration of fixations and dwells directed at another’s face, scanpaths (i.e., the order in which features of another’s face were scanned), and so on. For a comprehensive list of possible measures, we refer the reader to Holmqvist et al. (2011) or Holmqvist and Andersson (2017). 

With dual eye-tracking setups, the temporal and spatial relation between two gaze position signals can be analyzed. This allows for various other measures of gaze behavior, such as those relating to mutual gaze (i.e., how often and how long people look at each other) or shared gaze (i.e., how much people direct their gaze at the same object during a task). Examples of mutual and shared gaze are given in the dual eye tracking section. In addition, cross-recurrence and cross-correlational analyses can be used to investigate the temporal and spatial relation between two gaze position signals in more detail (see e.g., Ho, Foulsham, & Kingstone, 2015 for cross-correlational analyses; and Richardson & Dale, 2005; Yu & Smith, 2013 for cross-recurrence analyses). Finally, measures such as time-based entropy can reveal whether the spatial distribution of gaze differs between various actions of the other participant (e.g., looking toward or away from another person, see Hessels, Holleman, Kingstone, Hooge, & Kemner, 2019 for an example). 

Note, however, that besides AOI-based measures, there are also other relevant eye-tracking measures. One could, for instance, obtain global measures such as the frequency of blinks or saccades, as was already illustrated in the example by Dowiasch et al. (2015). With such measures, data can often be analyzed with similar ease irrespective of what type of setup is used. For a more detailed consideration of all the types of eye-tracking measures that can be used we again refer the reader to Holmqvist et al. (2011) or Holmqvist and Andersson (2017), as well as to the specific studies mentioned in the following sections.

### Single eye-tracking setups

Practically, analyzing the gaze data of one participant is often simpler than analyzing how the gaze of one person affects or is related to the gaze of another person. If a question can be answered with eye-tracking data from only one participant, this can significantly reduce the experimental analysis required and make the overall design less complicated. Furthermore, single eye-tracking setups are easier to finance because they require less eye trackers.

Single eye-tracking setups have been used in various ways to study human interaction. In one example, Freeth, Foulsham, and Kingstone (2013) used a single eye-tracking setup with a wearable eye tracker to investigate the gaze behavior of participants in both live and prerecorded interview situations with direct and averted gaze, and found that participants were more likely to look at the face of the experimenter when they were being asked a question than when they were answering a question, that the gaze behavior of interviewees was affected by the experimenter’s gaze (direct vs. averted) in the live condition, and that participants with increased autistic traits looked less at the experimenter in the prerecorded condition. Freeth et al. were interested in the gaze behavior of interviewees. It was therefore not necessary to record the gaze of the interviewer to answer the research questions they had. In another study on the relation between direct gaze and autism spectrum disorder (ASD), Falck-Ytter (2015) had children diagnosed with ASD and typically developing children perform a short-term memory task with an experimenter while measuring the gaze behavior of the children with a remote eye tracker. The experimenter either gazed directly at the child or averted their gaze from the child. A third example is the study by Gredebäck, Fikke, and Melinder (2010), who had infants interact with either their mother or a stranger to investigate how their gaze following abilities develop and whether there is a difference when following the gaze of mothers versus strangers. The infants were seated across the table from a model (either their mother or one of the experimenters) who had two toys placed in front of her. The model shifted their attention to one of two toys in an unpredictable manner while the gaze following behavior of the infant was recorded using a remote eye tracker. In these three studies, the behavior of the interactive partner was scripted and always performed in a similar manner. The research interest was the gaze of only one of the interactive partners; it was therefore not needed to record the gaze of both.

 There are also examples where the researchers needed to know the exact timing of the scripted behavior. This was the case for Nyström, Bölte, Falck-Ytter, and The EASE team (2017), who scripted the behavior of a test leader while measuring the gaze of infants using a head-boxed setup to investigate whether infants at risk for autism showed differing responses to direct gaze. To answer their research questions, the timing of the scripted gaze events of the test leader had to be coupled with gaze data from the infants. To achieve this, the researchers manually coded the gaze behavior of the test leader from video recordings. This was possible to do without a second eye tracker, since they were only interested in crude estimations of the gaze of the test leader (i.e., whether the gaze direction was left, right, up, or direct). In another study with the same setup, Nyström, Thorup, Bölte, and Falck-Ytter (2019) measured the gaze of infants to see whether infants at risk for autism showed different patterns of gaze when responding to or initiating joint attention (i.e., episodes where both the infant and the parent attend to the same object). To create scenarios where initiating and responding to joint attention could be analyzed, the gaze behavior of the test leader was again scripted and coded manually, as fine-grained analysis of the gaze of the test leader was not necessary to answer the research questions (see Thorup et al., 2016, 2018 for related studies using the same setup).

If an interaction experiment is designed in the way that the gaze behavior of one participant is not necessary for the research question or is scripted, a single eye-tracking setup can suffice. However, if the gaze (or other behavior) of the scripted participant is used by the researchers in some way, some form of manual coding may be necessary. When the behavior of one of the interactants in an experiment is scripted, it may be necessary to manually confirm that the behavior was conducted according to the script. Another way to accomplish this is to use eye tracking. We will return to this point in the dual eye-tracking section. The examples mentioned above thus illustrate how single eye-tracking setups allow researchers to investigate important questions regarding human interaction by tracking the gaze of one of the interacting agents. It is important to keep in mind that other methods to estimate gaze (e.g., video cameras and manual coding) can be used to complement single setups. With such methods, it can be possible to analyze the gaze signal provided by the eye tracker in relation to the gaze behavior of the other interactant.

### Dual eye-tracking setups

Interaction, as we have defined it, is action occurring between (at least) two individuals. An interactive situation will thus always involve two or more participants who do not act independently. Their actions can both influence and be influenced by each other. With eye trackers to record the gaze behavior of both participants engaged in interaction, the extent of how the gaze of one participant might influence the other or how the gaze behavior of two interactants are more broadly related can be captured in greater detail than by using a single eye tracker.

One example of a dual eye-tracking study is given by Macdonald and Tatler (2018), who measured the gaze of participant dyads using wearable eye trackers. The dyads were instructed to work together to make a cake in a kitchen environment. The authors showed that participants spent very little time looking at each other during the task, and that dyads who were assigned predefined roles (i.e., chef and gatherer) looked at each other more and were faster to align their gaze to the same object than dyads without predefined roles. This study demonstrates the general motivation for dual eye tracking: to measure how the gaze behavior of two individuals relate to each other. With dual eye tracking, it is possible to operationalize variables relating to concepts such as mutual gaze and shared gaze.

Joint attention is often a variable of interest in eye-tracking research and is considered an important factor in early social development (Frischen, Bayliss, & Tipper, 2007; Nyström et al., 2019). This makes shared gaze particularly interesting for research on parent–child interaction. In a relevant study on this topic, Yu and Smith (2016) equipped 11- to 13-month-old infants and their parents with wearable eye trackers. They then measured periods of joint attention (i.e., periods where the infant and parent jointly fixated on the same object) and sustained attention (i.e., periods where the infant fixated on an object). By comparing periods of joint attention to periods of sustained attention, they found that when parents fixated objects infants were already fixating, the infants were more likely to keep fixating those objects longer. A common theme in dual eye-tracking studies is that the research questions addressed in them primarily concern the synchrony of gaze events, such as how often participants look at each other simultaneously, how often they look at the same object simultaneously, or where one participant looks when the other is performing some action, e.g., talking to or looking at the other participant. These examples highlight how dual eye-tracking setups are ideal to examine the dyadic nature of interaction.

Using a dual eye-tracking setup might also be necessary or beneficial even in experiments where the gaze behavior of one of the interactants has been scripted. An example of this can be seen in the second experiment reported in Hessels, Cornelissen, Hooge, and Kemner (2017), where the researchers manipulated the gaze behavior of a confederate and examined whether it had an effect on the gaze behavior of the participant. The confederate was instructed to gaze at the participant in a specific way. However, it was important to confirm that the gaze behavior of the confederate had occurred according to script. To achieve this, the researchers used a dual eye-tracking setup to measure the gaze of both the participant and the confederate. An additional eye tracker can therefore be used as an objective tool to validate the scripted gaze behavior.

Adding an eye tracker to a setup can result in some notable difficulties. As we have illustrated, researchers using dual eye-tracking setups have been primarily concerned with the synchrony of gaze events between participants. However, this means that one needs be able to relate the gaze position of one participant at a specific timepoint to the gaze position of the other participant at the *same* timepoint. This can be done, for example, by using a common timestamp for the time series of two participants.

Multiple eye trackers in a setup will likely result in other technical difficulties as well. An eye tracker, regardless of type, is typically connected to a computer during at least some point of the recording. Connecting multiple eye trackers to one computer might cause conflicts with eye-tracker software and using an additional computer with each additional eye tracker requires a larger financial investment. Another factor to consider when adding an eye tracker to a setup is the economic burden that comes with it. High-end eye trackers are priced in the tens of thousands of euros, while some of the more low-end eye trackers can be bought for a few thousand euros per piece (see e.g., Hessels, Cornelissen, Kemner, & Hooge, 2015). Doubling the budget for eye trackers might not be an option in many cases. In sum, an additional eye tracker will generally make the setup more difficult to build and to operate.

### Solutions to the synchronization problem

In order to synchronize different eye trackers, researchers often use external events (such as briefly flashing a light or playing a sound) to mark the start and/or end of an experiment. The timestamp of such an event can then be used to relate the data from the two separate systems to each other (see e.g., Broz, Lehmann, Nehaniv, & Dautenhahn, 2012; Yu & Smith, 2016 for examples). Another potential way to deal with this problem is to have all the relevant components of a setup run from the same computer. In this manner, each component reports data using the same system clock, eliminating the need for any further synchronization. However, this is not always possible. In many cases, dedicated software is required to run one eye tracker. Recording from two eye trackers simultaneously is not always within the capabilities of the software. Furthermore, wearable eye trackers are often built to be carried around and thus are not connected to a computer during recording.

Yet another solution to the synchronization problem is to use one central computer with a “master clock” and have it send synchronization signals to the computers recording from the eye trackers (e.g., using a parallel port or TCP/IP). The implementation of this may require building customized hardware and software. In the case of screen-based setups, additional steps are needed to minimize the delays from the recording of a webcam to the presentation of the video image on a screen.

The solutions mentioned above, such as flashing a light or playing a sound, require less technical skills but more manual labor as these events need to be manually identified from various recordings. It is of course possible to try to automatize the coding of a flashing light or presentation of a sound; yet, this requires at least some sort of algorithm to be developed. In short, there are no easy solutions to the synchronization problem.

## Choice of eye tracker

The choice for the type of setup is not simply a question of preference, as they differ in various characteristics. Here we focus on the three key characteristics important for research on human interaction: freedom of movement for the participant, eye-tracking data quality, and ease of data analysis. Although we choose to focus on these three characteristics, other factors, such as the sampling rate of the eye tracker and whether gaze is measured monocularly or binocularly, are also important for determining what questions can be asked with eye-tracking research in general. We refer the reader to Andersson, Nyström, and Holmqvist (2010) for a discussion of the effect of sampling rate on eye-movement measures and Hooge, Holleman, Haukes, and Hessels (2019) for a discussion on binocular vs. monocular eye tracking.

**Freedom of movement** refers to how restrictive the eye tracker is in terms of constraining head, limb or body movement. **Data quality** refers to the reliability, validity, and availability of the eye-tracking data. It can be expressed with the measures of precision, accuracy, and data loss. Precision is an indicator of how reliable the eye-tracking data is. When the same area of the world is continuously looked at and the eye tracker reports gaze coordinates that are very close to each other, precision is high. Accuracy, on the other hand, is a measure of validity. If gaze coordinates reported by the eye tracker are close to the actual point the participant looked at, accuracy is high. Data loss refers to the amount of gaze samples lost due to e.g., the eye tracker not being able to detect the pupil or the CR (Holmqvist, Nyström, & Mulvey, 2012). Together, these three measures describe the quality of the eye-tracking data. **The ease of data analysis** describes how easy it is to analyze the data from the eye tracker and is highly dependent on the type of measures used. Our focus will be on the ease of data analysis with respect to AOI-based measures as they are common in eye-tracking research on human interaction as well as in eye-tracking research in general. AOIs are essential when one wants to draw conclusions about where one looks at another and for how long, i.e., whenever gaze needs to be mapped to the visual world. Table 2 illustrates how the different types of eye-tracking setups used in the interaction literature compare to each other on these three criteria. In the following section, we will discuss the different types of eye-tracking setups with these criteria in mind along with the implications that the choice of eye tracker has on the research questions posed.

**Table 2:** Comparison of eye-tracking setups in relation to our evaluation criteria. The reported values have been adapted from eye-tracking studies using such setups (see e.g., Hessels & Hooge, 2019; Hessels, van Doorn, et al., 2020; Holmqvist, 2017; Niehorster, Cornelissen, Holmqvist, & Hooge, 2019; Niehorster, Santini, et al., 2020). Note that our discussion on the ease of data analysis is focused on AOI-based measures and might not apply to studies using global eye-tracking measures (e.g., fixation duration, saccade amplitude, blink rate, pupil size, etc.)

	Head-free	Head-boxed	Head-restricted
Freedom of movement	Head and body can move freely	Head and body can move within the head box	Head and body movement restricted
Typical data quality	Precision up to (variable error as low as) 0.5+° Accuracy up to (systematic error as low as) 1–3° Data loss 0–20%	Precision up to (variable error as low as) 0.1–0.3° Accuracy up to (systematic error as low as) 0.5–1° Data loss 0–20%	Precision up to (variable error as low as) 0.01–0.03° Accuracy up to (systematic error as low as) 0.5° Data loss 0%
Ease of data analysis	Difficult: often manual AOI analysis, point of view changes over time and is unique for each participant	Easy: often automatic AOI analysis	Easy: often automatic AOI analysis

### Head-free setups

Head-free setups typically contain one or more wearable eye trackers. The eye trackers in these setups are fixed to the head. As such, they report gaze coordinates relative to the head. This is different from the eye trackers found in head-boxed and head-restricted setups, which report gaze coordinates fixed to the world. When a participant fitted with an eye tracker in a head-free setup moves their head, the coordinate system moves together with the head. Recent technical developments have made eye tracking available for virtual and augmented reality as well. Combined with eye tracking, these new technologies provide interesting opportunities for designing setups where participants interact with each other in virtual or augmented reality. Virtual and augmented reality systems typically take the form of goggles or glasses that are mounted on the head and can be considered to follow the same principles as head-free setups. At first glance, it might make sense to use head-free setups as the primary choice to investigate human interaction, as they allow the user to freely engage with the environment (see Franchak, 2020; Pérez-Edgar, MacNeill, & Fu, 2020 for further discussion of the possibilities of wearable eye tracking). This is reflected in the number of interaction studies with head-free setups compared to studies with head-boxed and head-restricted setups (see Table 1). Wearable eye-tracking technology, however, is still far from optimal, and wearable eye trackers are often not as reliable and accurate as advertised (see Hessels, Niehorster, Holleman, Benjamins, & Hooge, 2020 for a discussion).

#### Freedom of movement

The greatest advantage of the eye trackers in head-free setups is the freedom they provide. Unlike the eye trackers used in head-boxed and head-restricted setups, the eye trackers in head-free setups typically do not need to be connected to a computer with a wire but can be carried on the participant (e.g., with a mobile recording unit attached to eye-tracking glasses such as with the Tobii Pro Glasses 2). Because of this, head-free setups with wearable eye trackers are ideal for designing experiments that require participants to be in motion. Consider the study by Macdonald and Tatler (2018), where participants were tasked to bake a cake with each other. In this type of a situation, it makes intuitive sense to use a head-free setup; cooperating with each other to bake something is generally a task which requires quite a bit of freedom of movement. It would likely prove difficult to have two participants cooperate on a baking task if they were restricted in how they can move parts of their body, such as the head and hands, or if something were in the way (e.g., if there was a remote eye tracker placed in front of each participant). It could be argued that the freedom of movement that head-free setups provide was necessary to complete the task. In another study, Franchak et al. (2011) equipped infants with wearable eye trackers and had them engage in free play with their mothers. The researchers were interested in infants’ gaze behavior during unrestrained interaction. They showed that infants did not look at their mothers’ faces often during the free play session but did so when the mothers were sitting at their level. The authors concluded that the way infants scanned the environment was largely dependent on the information available as well as on the constraints of their own bodies. The study further emphasizes how the choice of eye tracker can be utilized to design experiments that are highly representative of the behavior they are used to investigate. The researchers were interested in infant gaze during unrestrained interactions where infants were free to choose their own body posture; it would therefore not be representative of the situation to have the infants seated in a head-boxed setup with a remote eye tracker. A third demonstration of how freedom of movement can be applied in interaction research is given by Franchak et al. (2018), who measured the gaze of infants and parents while they were engaged in free play together. The experiment required that both parents and infants were able to move around the room and play with toys. Thus, it could only be conducted using wearable eye trackers.

#### Data quality

The eye trackers in head-free setups generally provide less spatially accurate and precise data than eye trackers in head-boxed and head-fixed setups. This can further be exacerbated by movements of the eye tracker with respect to the head, as was evident in a study by Niehorster, Santini, et al. (2020), who fitted participants with wearable eye trackers and measured their gaze while they performed actions that caused the eye-tracking glasses to move, such as speaking or making facial expressions. The results revealed that even small amounts of movement in most (but not all) commercially available wearable eye trackers resulted in significantly reduced data quality. It is therefore not surprising that the majority of the interaction studies using wearable eye trackers included in this review did not distinguish between facial features and instead chose to make cruder distinctions, such as whether gaze was directed on the face (or object) or somewhere else (Broz et al., 2012; Damm et al., 2013; Franchak et al., 2011; Freeth et al., 2013; Fu, Nelson, Borge, Buss, & Pérez-Edgar, 2019; Gullberg & Holmqvist, 1999, 2006; Hanna & Brennan, 2007; Ho et al., 2015; Macdonald & Tatler, 2013, 2018; Nadig, Lee, Singh, Bosshart, & Ozonoff, 2010; Yamamoto, Sato, & Itakura, 2019; Yu & Smith, 2013, 2016; Yu, Suanda, & Smith, 2019). Five of the reviewed studies with wearable eye trackers distinguished between gaze directed at different regions of the face: Cañigueral, Hamilton, and Ward (2018) and Cañigueral, Ward, and Hamilton (2020) divided the face into an eye region and a mouth region and Haensel et al. (2020) divided the face into an upper and lower region, while Rogers, Speelman, Guidetti, and Longmuir (2018) and Freeth and Bugembe (2019) further differentiated between the finer details of the face (i.e., eyes, nose, mouth, etc.).

#### Ease of data analysis

Head-free setups can be problematic when considering the analysis of the eye-tracking data. This is mainly because the eye trackers in these setups are usually equipped with a scene camera that moves together with the head. To find out where people look at in the world, one needs to know what is visible in each frame of the scene camera recording, and where gaze is located with respect to it. This is difficult, since the scene camera image is constantly changing as a participant moves through the world. In head-boxed eye tracking, it is often known what is visible to the participant at each point in time, because the experimenter presents it. As such, it is easy to determine where one looks with respect to the visual stimulus, for example by using an area-of-interest (AOI) method. However, generating AOIs on a constantly moving scene is a difficult task to automate. Because of this, most researchers using head-free setups resort to manual coding when generating AOIs and mapping gaze data to the world (Benjamins, Hessels, Hooge, 2018). This is highly disadvantageous as manual coding is extremely time consuming, which is particularly evident in setups with multiple eye trackers. Another problem with the eye trackers in head-free setups is that the eye trackers themselves can affect the gaze behavior of others. As reported by Cañigueral et al. (2018), people might show altered gaze behavior when interacting with a person fitted with a wearable eye tracker. This type of interference could potentially lower the representativeness of interactive situations where head-free setups are used.

### Head-boxed setups

Head-boxed setups are typically equipped with one or more remote eye trackers. The eye trackers in these setups are used without restricting the head and are typically placed on some platform at a fixed distance in front of the participant (see Fig. 1b). These types of eye trackers are stationary and provide gaze data in world-centered coordinates. This means that the coordinate system of the gaze position is fixed to the world and does not depend on head orientation or position. In other words, even if the participant looks at the same point on a computer screen and moves their head slightly to either the left or the right, the gaze coordinates reported by the eye tracker should not change. The eye trackers in head-boxed setups can be used either with screens, in which case participants look at each other through monitors similar to an online video call, or without screens, meaning that there is nothing in between the participants except for the eye trackers. In the latter case, a view of what each participant is looking at is usually extracted from video recordings using separate cameras (see e.g., Falck-Ytter, 2015; Gredebäck et al., 2010; Nyström et al., 2017, 2019; Thorup et al., 2016, 2018 for examples of head-boxed eye-tracking setups without screens).

Not using screens can be advantageous to using screens in some situations. Having participants seated across each other face-to-face, without screens, is arguably often more representative of a typical face-to-face conversation that a person might have on a regular day (although the COVID-19 pandemic might be changing this for many people). Furthermore, not having screens in between participants makes certain physical interactions feasible (as in head-free setups). For instance, if one participant would need to hand over an object to the other participant, a screen would be in the way.

**Fig. 2:**
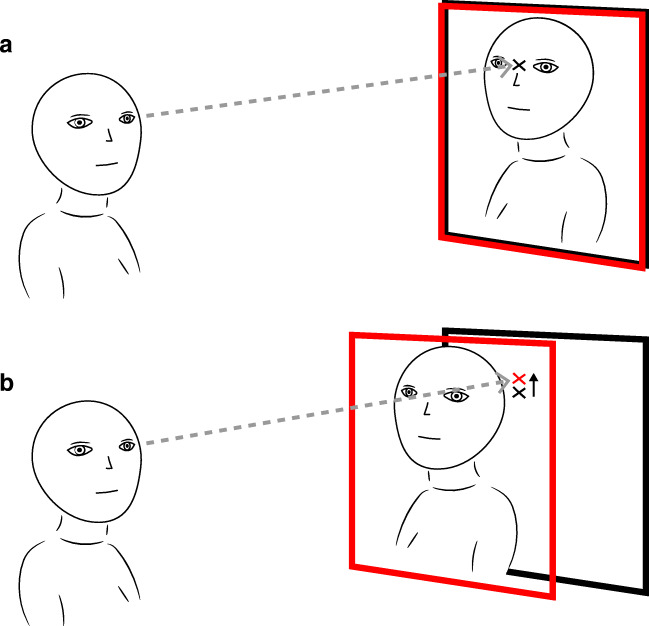
The parallax error in a head-boxed setup without screens. The *black frame* indicates the plane to which the eye tracker is calibrated. This means that the eye tracker reports a gaze position in the black plane. The *red frame* indicates the plane of regard, i.e., the plane to which the observer looks. **a** The plane of regard and the calibrated plane roughly coincide. The eye tracker accurately reports where the observer looks. **b** The plane of regard does not coincide with the calibrated plane (in this case, it is moved toward the observer). This causes a relative offset (here an upward shift indicated by the *black arrow* in panel **b**) in the reported gaze position compared to the situation when the calibrated plane and plane of regard coincide

A potential disadvantage of head-boxed setups without screens is the possible parallax error that may be observed (see Fig. 2). For the eye tracker to accurately report where someone looks on the face or body of another, that person would have to remain within the calibrated plane of the eye tracker (the black frame in Fig. 2). This can become problematic when people interacting with each other do not sit still. If, for example, one moves forward or backward such that they are no longer within the calibrated plane while the other keeps looking at one's eyes, this will result in a systematic parallax error in the gaze-position signal of that other person. This is not the case with a head-boxed setup with screens, since even if a person moves forward or backward, (s)he is still projected to the same calibrated plane for the other person.

#### Freedom of movement

The eye trackers in head-boxed setups allow for movement of the head; however, they commonly operate within an area called the “head box” (i.e., the range of allowed head movement). These eye trackers are capable of reporting gaze coordinates as long as the head stays within the predefined head box. The size of the head box varies between eye tracker models. A general limitation of head-boxed setups is that although the head is technically free to move to some extent within the head box (depending on the eye tracker model used), they are still quite restrictive. Participants are generally instructed to try to sit as still as possible. It would therefore be impossible to replicate e.g., the experiment by Franchak et al. (2011) using a head-boxed eye-tracking setup, since the infants would not be able to engage in unrestricted free play and would instead have to remain still with an eye tracker placed in front of them. Head-boxed setups might be more suited if one were to investigate the dinner table gaze behavior of infants, as they are commonly seated in restrictive baby chairs during such situations.

#### Data quality

An important advantage of head-boxed setups compared to head-free setups is the increase in the quality of the eye-tracking data provided, which allows for analysis of the more fine-grained details in gaze behavior, such as which region of the face is being looked at. The greater precision and accuracy of eye trackers typically used in head-boxed setups is demonstrated by Merin, Young, Ozonoff, and Rogers (2007), who investigated whether infants who had siblings with ASD showed different gaze behavior than comparison infants when reacting to a modified “still face” paradigm (i.e., a paradigm where there was first normal interaction between the mother and infant, followed by a sudden period of the mother being completely expressionless, after which normal interaction resumed again). Merin et al. differentiated between gaze to the eyes, mouth, and other face regions, and found that there was a subgroup of infants who looked less at the eyes of the mother relative to the mouth when the still face was presented.

The possibility for more fine-grained distinctions between regions of the face was further utilized by Hessels et al. (2017), who used a dual eye-tracking setup to investigate whether there was a bias for attending the eyes when interaction is possible, and whether the gaze pattern of one partner in an interactive situation might affect the gaze of the other partner. Hessels et al. differentiated between gaze directed at the left eye, right eye, nose, and mouth, and found that participants looked longer at the other participant’s eyes when compared to other parts of their face. These examples, as well as other interaction research done with head-boxed setups show how they can be used to look at gaze behavior with greater resolution compared to wearable eye trackers (Haith et al., 1977; Hessels et al., 2019; Hessels, Holleman, Cornelissen, Hooge, & Kemner, 2018; Merin et al., 2007; von dem Hagen & Bright, 2017).

#### Ease of data analysis

Another advantage of head-boxed setups is that they facilitate the use of automatic analysis software more so than head-free setups. Mapping gaze to the world is easy as the visual stimulus can be described in the same coordinate system as the gaze position reported by the eye tracker (in contrast to wearable eye tracking where the coordinate system itself moves). An example of this can be seen in Hessels et al. (2019), where gaze data was automatically mapped to the faces of the interacting partners using automatically generated regions of interest. While head-boxed eye-tracking setups might not be suited to investigate gaze behavior during locomotion or even during tasks that require some amount of head and/or torso rotation and movement (e.g., cooperating to bake a cake), their advantages become evident in situations where people are mainly talking with each other in a relatively still position.

### Head-restricted setups

Similar to head-boxed setups, the eye tracker(s) in head-restricted setups are usually placed in front of the participant. With tower-mounted eye trackers this might not be the case. However, we are not aware of interaction studies using tower-mounted eye trackers. The striking feature of head-restricted setups is that the head is fixed in place using some apparatus, such as a chin rest and/or a forehead rest. Using only a forehead rest may allow the participant some additional freedom (e.g., the possibility to talk during measurement). The coordinate system in this case is fixed to both the world and the head.

#### Freedom of movement

The most notable limitation of head-restricted setups is their restrictive nature. Fixing the head in place is beneficial for data quality: when the head cannot move, the availability of the pupil and the CR for the eye tracker is generally much better than when the head can move. In other words, restricting head movement minimizes the chance of the eye tracker losing signal of the eye. However, because of this, head-restricted setups have not been used in many interaction studies; fixing the head in place might not allow interactive behavior that is representative for human interactive situations. In practice, this will make studies with infants or certain clinical populations impossible, and studies were freedom of facial movement is required (facial expressions, talking, etc.) impractical.

#### Data quality

The advantages of head-restricted setups are the same as with head-boxed setups; however, the additional restriction of head movement brings with it an increase in data quality. Head-restricted setups are therefore ideal to investigate interactive situations in which head movement is typically not involved or does not matter, and/or when measuring the more fine-grained details in gaze patterns. Holleman, Hessels, Kemner, and Hooge (2020), for example, used a head-restricted setup to investigate whether and how the potential for interaction affected gaze to faces, specifically how participants looked at the eyes of a confederate. Participants viewed prerecorded clips of confederates, with the twist that half of the participants were led to believe that they were interacting with another person through a live camera setup, while the other half were led to believe that the clips were prerecorded. Holleman et al. used a chin and forehead rest to minimize head movements and to keep the viewing conditions between participants constant. They found that although gaze behavior to the faces of confederates varied greatly between participants irrespective of condition, a subset of participants in the live condition were less likely to gaze at the eyes of the confederate compared to participants in the prerecorded condition. In a similar study by Nakamura, Kamiya, and Yoshida (2017), participants viewed each other’s faces through a live video feed, prerecorded videos, and static image presentations. Nakamura et al. wanted to know how well participants were able to judge whether they were viewing their interactive partner in real time and whether there were differences in gaze directed at the eye region between live and non-live conditions. Participants were placed in a chin rest in front of a remote eye tracker, viewing each other through computer screens. The authors found that when looking at a live video feed, participants gazed less at the eye region of their interactive partner compared to when looking at pre-recorded videos. These studies illustrate how head-restricted setups can be utilized: if allowing the head to move freely during an experiment is not necessary to answer the question, it can be useful to lock the head in place to maximize data quality and to keep the viewing conditions between participants and/or conditions constant.

#### Ease of data analysis

Head-restricted setups deliver optimal eye-tracking signals for signal processing and data analysis. Both the head and the eye tracker itself is fixed to the world, providing the eye tracker optimal conditions to detect the pupil and CR. The same advantages that head-boxed setups have in terms of automatic data analysis apply here as well. Holleman, Hessels, et al. (2020) made use of these advantages by employing an open-source algorithm to automatically detect faces and facial landmarks in stimulus clips and used these to generate AOIs (see Hessels, Benjamins, Cornelissen, & Hooge, 2018 for a validation of the method). Using the algorithm, they were able to determine the position of the eyes, nose, and mouth for every frame of the prerecorded videos they used. Using methods such as these can greatly reduce the time and financial investment (i.e., in the salaries of manual coders) required to analyze the eye-tracking data.

### Eye-tracker models and software

Although head-free, head-boxed, and head-restricted setups differ on average in e.g., data quality (as the values in Table 2 illustrate), reality is slightly more complicated than what we just described. Within each category there are eye trackers that may outperform those in other categories. The eye trackers at the high end of the price spectrum generally outperform the ones at the low end. The range of the price spectrum, however, differs quite widely between categories. The eye trackers at the high end of the head-boxed and head-restricted categories tend to be more expensive than the ones at the high end of the head-free category. The choice for a particular eye tracker model is often a trade-off between the capabilities of the eye tracker and the financial burden imposed on the researcher. We will provide two examples to illustrate this choice.

Consider first an example experienced by one of the authors: to build a dual eye-tracking setup with remote eye trackers and screens, an eye tracker that could be used with an analog trigger to minimize potential delays for starting and stopping a recording was needed. Two eye trackers capable of this were considered: the SMI RED and the Tobii TX300. The technical specifications of both eye trackers in terms of data quality and sampling frequency were good enough for the planned setup. However, the SMI RED was cheaper and therefore the more accessible option. Sometimes, such a choice might not be available. This was the case for Franchak et al. (2011), who wanted to investigate the looking behavior of infants in situations where they could move around freely. At the time, no eye trackers with such capabilities were available, leading the researchers to collaborate with the eye-tracking company Positive Science to develop the first wearable eye tracker specifically designed for infants.

These examples show that the choice of eye tracker is always dependent on what is required and what is available. To choose the optimal eye tracker model for an eye-tracking setup, we recommend to first define the requirements for the setup, then consult any relevant literature. Niehorster, Santini, et al. (2020), for example, compared how slippage affects the data quality of wearable eye trackers and found that some models greatly outperformed others. These types of studies can help researchers interested in head-free setups choose the most suitable eye tracker model.

Still another matter to consider when choosing an eye tracker for a setup is the choice of eye-tracker software. Eye trackers are typically sold with manufacturer software. These software packages contain tools for measuring gaze, designing simple experiments, extracting data, and even for performing certain analyses (e.g., fixation or saccade classification). Many of the manufacturer options, however, are quite limited in their capabilities. Starting measurements from two eye trackers at the same time, for instance, often requires one to deviate from the stock options and work with programming languages and software development kits instead. Several alternatives have been developed to expand on what stock software packages have to offer. One example is the open-source toolbox called GazeCode, which allows the user to classify fixations in data from wearable eye trackers and map these to the visual stimulus more efficiently than with manufacturer software (Benjamins et al., 2018). Another example is GlassesViewer, which is open-source software for viewing and analyzing data from the Tobii Pro Glasses 2 wearable eye tracker (Niehorster, Hessels, & Benjamins, 2020). Alternative software for eye trackers gives more flexibility for researchers but can at the same time require programming skills.

## Choosing the right setup

**Fig. 3:**
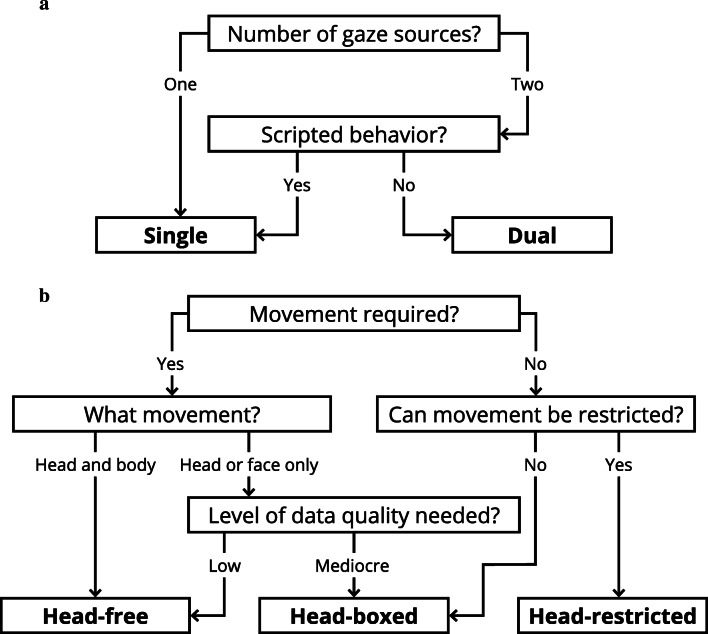
A decision tree to make some of the choices for selecting the right type of setup explicit. Part **a** represents the choice of a single versus a dual setup, while part **b** represents the choice of type of eye-tracking setup. Of course, it is a schematic overview and not all possible choices are present. For a more nuanced consideration of the different eye-tracking setups, we refer the reader to the main text

Now that we have introduced the types of eye-tracking setups used in human interaction and discussed their possibilities and limitations, we are faced with the problem of choosing the optimal setup. This problem is relevant for anyone interested in investigating gaze behavior during human interaction, particularly for those starting out in the field and those who are not yet familiar with the technology. We have distilled our discussion down to the decision tree depicted in Fig. 3. The decision process is divided into two parts, the choice of using a single vs. a dual setup followed by the choice of type of eye-tracking setup.

Eye-tracking setups are commonly built keeping some general line of inquiry or research idea in mind. Therefore, it is vital to understand the limits of the setup and confine the research questions within those limits. The first part of our decision scheme is the choice of a single versus dual setup (see Fig. 3a). Single eye-tracking setups can be used to investigate many relevant aspects of human interaction but are ultimately restricted to the gaze data of one person. Analyzing the relation between the looking behavior of two people requires a dual eye-tracking setup, unless one uses different methods to estimate the gaze of the other person, e.g., by manually coding from video. However, in certain situations the need for gaze data from two people can be circumvented by scripting the looking behavior of the other person in advance.

The second choice involves the type of eye-tracking setup (see Fig. 3b). This choice boils down to the trade-off between the three characteristics discussed in the previous sections: freedom of movement, data quality, and the ease of data analysis. Research questions that require movement typically favor head-free setups, whereas optimal data quality and the need for automatic data analysis are likely to favor head-boxed and head-restricted setups (although there have been advancements in automatic methods for detecting AOIs with wearable eye tracking, see e.g., De Beugher, Brône, & Goedemé, 2014). Furthermore, in some situations restricting movement might be desirable but not ethical, such as in research with young children or certain patient populations.

To summarize, the number of gaze sources, use of scripted behavior, and the desired freedom of movement, data quality, and ease of data analysis are all factors that influence the choice of setup. By carefully considering what is needed to answer the research question and being aware of the limitations of the different types of setups, the optimal setup may be chosen.

## Future directions

Before we conclude, we briefly highlight several future directions that we believe will have an impact on the study of gaze during human interaction over the coming years and are thus important to be aware of.

As already discussed, automatic analysis of where participants looked in the world, both for eye-tracking studies using head-free setups and live eye-tracking studies using head-boxed and head-restricted setups, is highly desirable. In eye-tracking research in general, there have already been great advancements. Yet, in interaction research, automation has proved to be notoriously difficult, as it requires one to be able to automatically determine what a scene consists of. It is necessary to know where faces, body parts, and objects are located. Humans engaged in interaction rarely stay still; rather, they often move their heads and their bodies and even the objects around them. Automatically generating AOIs for non-static stimuli is challenging. Manual coding, on the other hand, can often be an extremely time-consuming process. Additionally, with manual processes it is difficult to rerun analyses with slightly different parameters, which is not the case for automatic methods. However, new methods for automatic generation of AOIs are constantly being developed, and some of these have already been validated (see Brône, Oben, & Goedemé, 2011; Hessels, Benjamins, et al., 2018 for examples). One might argue that automatic generation of AOIs is already quite possible using object- or person-detection algorithms. However, one should bear in mind that object detection is a necessary part of, but not identical with AOI-generation. One needs (1) to consider how far away from an object one can look and still perceive it (as discussed in Hessels, Kemner, van den Boomen, & Hooge, 2016), and (2) the accuracy of the eye-tracking data (see e.g., Orquin & Holmqvist, 2018). For example, in sparse environments, AOIs might be substantially bigger than the objects they encompass. As such, object- and person-detection algorithms are important first steps, but they need to be supplemented by dedicated AOI-methods (see e.g., Hessels et al., 2016) and validations of these methods using eye-tracking data (see e.g., Hessels, Benjamins, et al., 2018). AOI-methods can be validated, for example, by instructing participants to look at specific objects in a scene and then examining whether the method is capable of returning the same AOIs.

A recent innovation that is relevant for the study of human interaction through gaze behavior is appearance-based gaze estimation (see Zhang, Sugano, Fritz, & Bulling, 2015 for an introduction). Appearance-based gaze estimation is, as the name implies, estimating gaze direction based on the appearance of e.g., one's eyes or head from video recordings. There have been considerable developments in appearance-based gaze estimation, and many new algorithms and software packages are freely available. One example is OpenFace, an open-source appearance-based gaze estimation algorithm capable of facial landmark detection, head pose estimation, and gaze estimation (Baltrušaitis, Robinson, & Morency, 2016; Baltrušaitis, Zadeh, Lim, & Morency, 2018). The advantage of appearance-based gaze estimation is that it does not require a sizeable investment in dedicated eye-tracking hardware. Moreover, it can be used on video recordings, which can be acquired in many different settings. For example, it can be useful for research with participants for whom wearable eye trackers might be distressing (e.g., certain clinical populations), or in cases where a remote eye tracker placed in front of the participant would interfere with the experiment. In the context of human interaction, appearance-based gaze estimation could be used as follows: one could, for instance, use two web cameras each directed at one of the interacting persons. An appearance-based gaze estimation algorithm could then be applied to the movies and gaze direction of the participants can be estimated.

A major disadvantage of appearance-based gaze estimation methods is that, at present, the accuracy obtained with them is much lower than what is obtained with dedicated eye-tracking techniques. In their evaluation of person-independent 3D gaze estimation on two challenging datasets, Zhang, Sugano, Fritz, and Bulling (2017) reported an accuracy of 4.8 and 6.0 degrees. This is about ten times higher than with dedicated eye trackers, which can obtain accuracies of 0.5 degrees. As algorithms improve in the coming years, they may begin playing a more important role in the study of gaze during human interaction.

Another relevant topic is the co-recording of eye-tracking data with e.g., electroencephalography (EEG), functional magnetic resonance imaging (fMRI), electromyography (EMG), electrocardiography (ECG), or galvanic skin response (GSR). Some notable examples of studies where eye tracking is combined with another measure are by Wieser, Pauli, Alpers, and Mühlberger (2009), who used eye tracking together with both ECG and GSR to find out how direct and averted gaze are related to measures of physiological arousal, and Schilbach et al. (2010), who devised a method to measure gaze during fMRI recordings to investigate the neural correlates of joint attention. Although such co-recordings may be attractive from the perspective of combining different measures to understand human behavior in interaction, they come with additional challenges. One major challenge for co-recordings is that combining signals from different sources is often a complicated task that requires expertise in both methods (see e.g., Cornelissen, Sassenhagen, & Võ, 2019). Another challenge is that optimizing a setup for one particular method (e.g., eye-tracking) might result in a non-optimal setup for the other method (e.g., EEG). With EEG, it is ideal that the participant keeps their eyes, head, and body still, as any movement will result in large artifacts in the EEG data (Plöchl, Ossandón, & König, 2012). Obviously, this is not desirable when one is interested in looking behavior during free-flowing interaction. Combining eye tracking with EEG will thus place heavy limitations on the questions that can be asked with the setup. Similar trade-offs apply to other combinations of methods. Building and optimizing an interaction setup is an art in itself; adding more sensors or recording devices is likely to result in the setup not being optimized for any particular recording technique.

## Conclusions

We have presented a brief introduction to eye tracking as a technique and shown how the different types of eye trackers can be incorporated into setups used to investigate human interaction. Following, a scoping overview of the eye-tracking literature in human interaction was provided and several studies were reviewed and categorized depending on the amount and type of eye trackers used. We have furthermore examined how single and dual setups have been differently utilized in previous research and how three types of eye-tracking setups differ in the freedom of movement they allow, the eye-tracking data quality obtained, and the ease of data analysis. With these characteristics in mind, we have shown how the components and design of a setup place constraints on the research questions that can be answered about human interaction. To end the paper, we have provided a decision scheme to illustrate the process of choosing the right setup, intended for researchers planning to investigate gaze in the context of human interaction. We hope future research on this topic will make good use of our guidelines.

## Footnotes

1 Although we focus here on face-to-face interaction, i.e., when people can see or look at each other, there are also other kinds of eye-tracking studies involving interaction. Niehorster, Cornelissen, Holmqvist, and Hooge (2019), for example, measured the gaze of participants who either collaborated with each other or competed against each other in a visual search task. In another example, Richardson, Dale, and Kirkham (2007) investigated the gaze of participants who viewed images on a computer screen while conversing with each other through headsets.
